# Gene editing of the wheat homologs of TONNEAU1‐recruiting motif encoding gene affects grain shape and weight in wheat

**DOI:** 10.1111/tpj.14440

**Published:** 2019-07-28

**Authors:** Wei Wang, Qianli Pan, Bin Tian, Fei He, Yueying Chen, Guihua Bai, Alina Akhunova, Harold N. Trick, Eduard Akhunov

**Affiliations:** ^1^ Department of Plant Pathology Kansas State University Manhattan KS USA; ^2^ USDA‐ARS Hard Winter Wheat Genetics Research Unit Manhattan KS USA; ^3^ Integrated Genomics Facility Kansas State University Manhattan KS USA

**Keywords:** CRISPR‐Cas9, gene editing, grain size and weight, *TaGW7*, *Triticum aestivum*, wheat yield component

## Abstract

Grain size and weight are important components of a suite of yield‐related traits in crops. Here, we showed that the CRISPR‐Cas9 gene editing of *TaGW7*, a homolog of rice *OsGW7* encoding a TONNEAU1‐recruiting motif (TRM) protein, affects grain shape and weight in allohexaploid wheat. By editing the *TaGW7* homoeologs in the B and D genomes, we showed that mutations in either of the two or both genomes increased the grain width and weight but reduced the grain length. The effect sizes of mutations in the *TaGW7* gene homoeologs coincided with the relative levels of their expression in the B and D genomes. The effects of gene editing on grain morphology and weight traits were dosage dependent with the double‐copy mutant showing larger effect than the respective single copy mutants. The *TaGW7*‐centered gene co‐expression network indicated that this gene is involved in the pathways regulating cell division and organ growth, also confirmed by the cellular co‐localization of TaGW7 with α‐ and β‐tubulin proteins, the building blocks of microtubule arrays. The analyses of exome capture data in tetraploid domesticated and wild emmer, and hexaploid wheat revealed the loss of diversity around *TaGW7‐*associated with domestication selection, suggesting that *TaGW7* is likely to play an important role in the evolution of yield component traits in wheat. Our study showed how integrating CRISPR‐Cas9 system with cross‐species comparison can help to uncover the function of a gene fixed in wheat for allelic variants targeted by domestication selection and select targets for engineering new gene variants for crop improvement.

## Significance Statement

Grain size and weight are important components of a suite of yield‐related traits in crops. We used CRISPR‐Cas9 gene editing, gene expression network and diversity analyses to demonstrate that TaGW7: (i) affects grain shape and weight in wheat through pathways regulating cell division and organ growth; (ii) was the target of domestication and improvement selection; and (iii) can be used as a target for CRISPR‐Cas9‐based mutagenesis to expand diversity available for crop improvement.

## Introduction

Understanding the genetic basis of yield component traits in major crops holds great promise to improve yield potential by allowing breeders to make informed decisions while assembling beneficial alleles to create improved varieties. Yield component traits affecting grain size, shape and weight have been the subject of extensive genetic studies in a number of crops aimed to identify genes controlling these traits (Song *et al*., 2007; Takano‐Kai *et al*., 2009; Segami *et al*., 2012; Wang *et al*., 2012; Wang *et al*., 2015a; Xie *et al*., 2018; Wang *et al*., 2018b). Recently, several genes contributing to grain size and/or weight including *TaGW2* (2018a, 2018b), *TaCKX2* (Zhang *et al*., 2012a), *TaGASR7* (Zhang *et al*., 2016), *TaTGW‐7A* (Hu *et al*., 2016), and *TaGW8* (Yan *et al*., 2019) have been identified providing insights into the complexity of biological pathways that underlie regulation of grain size and weight in wheat. Many genes identified in wheat have rice homologs with similar function indicating that these developmental pathways are conserved among related grass species (Li and Li, 2016; Li *et al*., 2017).

The natural variation in the promoter of the *OsGW7/OsGL7* gene or its copy number in rice was shown to be associated with changes in grain morphology, grain quality and yield (Wang *et al*., 2015a,b). The highly expressed variant of this gene was linked with more slender grain, improved quality, reduced endosperm chalkiness, and higher yield, while the lowly expressed variant resulted in lower quality and shorter grain. *OsGW7* encodes a homolog of the TONNEAU1‐recruiting motif (TRM1) protein, a part of the complex involved in pre‐prophase band formation evolved to control oriented cell division during the formation of complex tissues (Hashimoto, 2015). Developmental pathways that affect morphological diversity of plant organs through the interaction with TRM appear to be common in plants. For example, it was shown that the interaction of the OVATE family proteins with TRM regulates cell division and alters fruit shape in tomato, and possibly in melon and cucumber (Wu *et al*., 2018). The high level of cross‐species conservation of pathways controlling grain morphology and weight (Li *et al*., 2017) suggests that the homologs of TRM in wheat might also be involved in biological pathways defining similar traits. Currently, the roles played by the *OsGW7* gene homologs in defining grain morphology and weight traits in wheat are not known.

In wheat, CRISPR‐Cas9 gene editing was successfully applied to modify such traits of agronomic value as disease resistance (Wang *et al*., 2014), gluten content (Sánchez‐León *et al*., 2018), protein content (Zhang *et al*., 2018), male sterility (Okada *et al*., 2019) and grain size and weight (Wang *et al*., 2018b; Zhang *et al*., 2018, 2019). These studies also demonstrated that the editing of either individual copies of duplicated genes on homoeologous chromosomes or all three homoeologous copies of genes is possible (2014, 2018a). For relatively low gene editing efficiency, mutants carrying editing events in multiple homoeologous gene targets can be obtained in the later generations of transgenic plants (Wang *et al*., 2018a). Here, we used CRISPR‐Cas9 gene editing to produce single‐ and double‐gene knockouts of *TaGW7*, and to study the effects of these mutations on grain morphometric and weight traits. To better understand the role of *TaGW7* in the regulation of biological pathways controlling these traits in wheat, we constructed the *TaGW7*‐centered gene co‐expression network. The evolutionary dynamic of the *TaGW7* gene region during wheat domestication and improvement was studied by analyzing the SNP diversity in a diverse panel of 818 accessions of hexaploid and tetraploid wheat (He *et al*., 2019).

## Results

### Identification of the *OsGW7* gene orthologs in wheat

**Figure 1:**
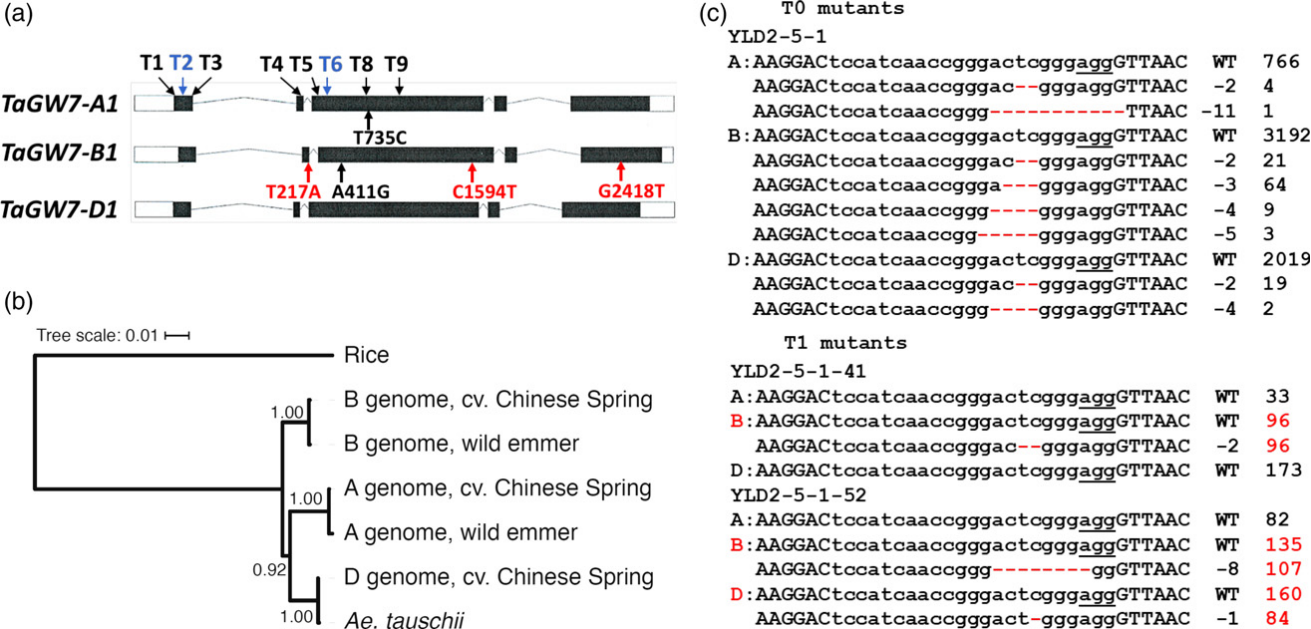
The structure, phylogeny and gene editing efficiency of the *TaGW7* gene homoeologs. (a) The exon−intron structure of the *TaGW7* gene homoeologs. The introns are shown as curved lines. The exons are shown as rectangles, in which open boxes correspond to the untranslated regions, and the filled boxes correspond to coding regions. Synonymous (T735C, A411G) and nonsynonymous (T217A, C1594T, and G2418T) mutations differentiating the respective *TaGW7* gene homoeologs between the Chinese Spring and Bobwhite cultivars are shown by arrows. The numbers correspond to the positions of mutated bases in the coding regions; black and red colors were used to designate synonymous and nonsynonymous mutations. Eight gRNA target regions conserved between at least two homoeologs of *TaGW7* are labeled T1–T6, T8, T9. The blue arrows correspond to targets selected for producing gene‐edited wheat lines. (b) Phylogeny of the *OsGW7* gene and its orthologs in wheat (cv. Chinese Spring), wild emmer and *Ae. tauschii*. The bootstrap values are shown at the tree nodes. (c) The aligned NGS reads flanking the GW7T6 target site and their frequencies in T_0_ line YLD2‐5‐1, and its T_1_ derivatives are shown. WT stands for wild‐type alleles; ‘−’ signs and numbers represent the number of bases deleted. The frequency of each mutation type is shown on the right. The PAM sequences are underlined. The deleted nucleotides are shown with red dashed lines. The gene homoeologs, which had edited alleles transmitted to the next generation of transgenic plants are highlighted in a red color.

We identified three wheat orthologs of the *OsGW7* gene, TraesCS2A01G176000, TraesCS2B01G202300, and TraesCS2D01G183400, that will be referred to as *TaGW7‐A1*,* TaGW7‐B1*, and *TaGW7‐D1*, respectively. The *OsGW7* orthologs in the ancestral species, tetraploid wild emmer *Triticum turgidum* ssp. *dicoccoides* (TRIDC2AG022470, TRIDC2BG025740) and diploid goatgrass *Aegilops tauschii* (AET2Gv20367100) will be referred to as *TtGW7‐A1*,* TtGW7‐B1*, and *AetGW7‐D1*. The *TaGW7‐A1*,* TaGW7‐B1*, and *TaGW7‐D1* genes each has five exons (Figure 1a) and encode proteins of 958, 950, and 954 amino acids in length, respectively (Figure S1). Compared with TaGW7‐A1, a protein sequence encoded by *TaGW7‐B1* has six amino acids deleted between position 48 and 53, and two amino acids deleted between positions 506 and 507. TaGW7‐D1 differs from TaGW7‐A1 by one deletion, four amino acids long, between positions 50 and 53. In addition to these deletions, we found 32 amino acid differences (3.4%) between TaGW7‐A1 and TaGW7‐B1, 25 amino acid differences (2.6%) between TaGW7‐A1 and TaGW7‐D1, and 23 amino acid differences (2.4%) between TaGW7‐B1 and TaGW7‐D1. The phylogenetic analysis of the *OsGW7* gene orthologs from hexaploid wheat, wild emmer, and *Ae. tauschii* showed clustering of sequences into three groups, each including the *TaGW7* homoeologs along with the homoeologs from the ancestral species (Figure 1b).

We have re‐sequenced the *TaGW7* gene homoeologs in cv. Bobwhite, which was used for the CRISPR‐Cas9 gene editing. Intercultivar comparison revealed several single‐base mutations differentiating the *TaGW7* gene homoeologs between cultivars Chinese Spring and Bobwhite. The *TaGW7‐A1* had one synonymous mutation T735C, and *TaGW7‐B1* had one synonymous mutation A411G and three nonsynonymous mutations T217A, C1594T, and G2418T. These nonsynonymous mutations result in amino acid replacements C73S, R532C, and K806N (Figures 1a and S1). The *TaGW7‐D1* gene sequence was identical in both cultivars.

### CRISPR‐Cas9 editing of the *TaGW7* genes in wheat

In total, eight CRISPR‐Cas9 target sites were selected within the first three exons of the *TaGW7* gene (Figure 1a). Seven target sites were conserved among the three homoeologs, whereas the GW7T4 target was conserved only between *TaGW7‐B1* and *TaGW7‐D1*. The gene editing efficiency of each gRNA target was assessed by the transient expression of the pA9Cas9sg constructs in the wheat protoplasts and next generation sequencing (NGS) of each target site (Wang *et al*., 2018a). These analyses showed that most of the targets could be edited by the designed CRISPR‐Cas9 constructs. With the protoplast transformation efficiency of ~30%, target site GW7T6 showed the highest level of editing efficiency of 8.3%, followed by GW7T9 and GW7T2, which showed the editing efficiency of 1.2% and 1.1%, respectively. The gRNA target sites GW7T2 and GW7T6 were selected for *TaGW7* gene editing (Figure 1a, Tables S1 and S2).

Two constructs, pA9Cas9sg‐GW7T2 and pA9Cas9sg‐GW7T6, were pooled in equimolar proportions and co‐transformed with pAHC20 into the immature embryos of cv. Bobwhite. Out of 15 regenerated plants, nine plants carrying both Cas9 and at least one of the two gRNAs were further analyzed by NGS. Out of these CRISPR‐Cas9 positive plants, target site mutations were detected only in plant YLD2‐5‐1. The line YLD2‐5‐1 contained low frequency mutated reads at target site GW7T6 in all three homoeologs of *TaGW7* (Figure 1c). By analyzing 70 plants from the T_1_ generation of YLD2‐5‐1 line, two plants heterozygous for the edited variants of the *TaGW7* gene were detected; the remaining plants were null for the mutations. The T_1_ line YLD2‐5‐1‐41 carried mutations in the *TaGW7‐B1* gene, and line YLD2‐5‐1‐52 had mutations in both the B and D genome copies of *TaGW7* (Figure 1c). All detected mutations caused frame shifts in the coding sequence (Figure 1c).

### Effects of the *TaGW7* gene editing on grain size and weight

The effects of CRISPR‐Cas9‐induced mutations in the *TaGW7* gene on grain morphometric traits (grain width, grain length, and grain area) and thousand grain weight (TGW) were evaluated in the T_2_ generation plants from YLD2‐5‐1‐41 and YLD2‐5‐1‐52. Preliminary evaluation was performed using a small sample of plants including seven and 37 plants of YLD2‐5‐1‐41 and YLD2‐5‐1‐52 T_2_ lines, respectively. The comparison of TGW and grain morphometric traits between Bobwhite and mutant lines with a homozygous mutation in the *TaGW7‐B1* gene (genotype *AABBDD* vs. genotype *AAbbDD*, where ‘*b*’ corresponds to the mutated allele in B genome), showed significant increase in grain width in the mutants (Figure S2). Because of the limited number of plants with genotypes *AABBdd* and *AAbbdd*, we compared phenotyping data collected for plants with the *AABbdd* and *AABbDD* genotypes to estimate the effects of mutations in *TaGW7‐D1*. This comparison showed significant decrease in grain length in the plants homozygous for mutation in *TaGW7‐D1* (Figure S2). The phenotypic effects of mutations in *TaGW7‐B1* and *TaGW7‐D1* were further evaluated in a small population of plants from the T_3_ generation of YLD2‐5‐1‐52 (Figure S3). In this population, the homozygous *TaGW7‐D1* mutants showed significantly increased grain width compared with that of the homozygous and heterozygous *TaGW7‐B1* mutants (Figure S3). The grain length in the *TaGW7‐B1* and *TaGW7‐D1* double‐gene mutants (genotype *AAbbdd*) was significantly decreased compared with that of the *TaGW7‐B1* mutants (genotype *AAbbDD*) (Figure S3).

**Figure 2:**
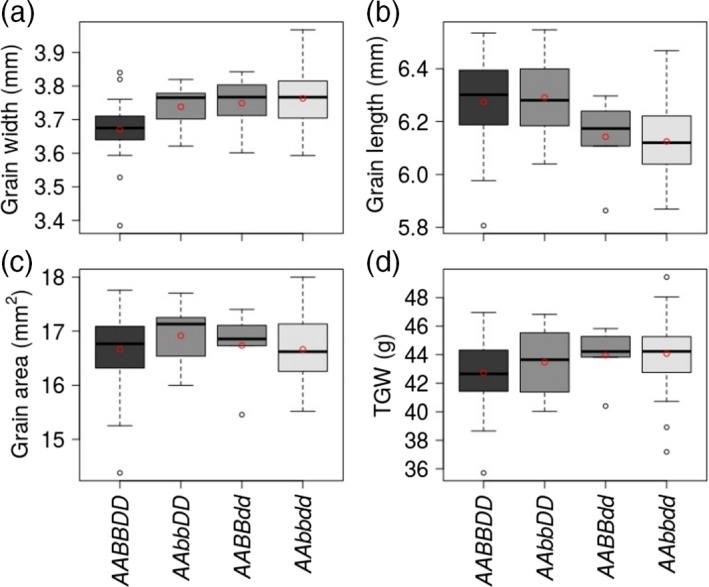
The effects of single‐genome and double‐genome mutations in the *TaGW7* gene on the grain morphometric and TGW traits in cv. Bobwhite. Box and whisker plots show the distribution of grain width (a), grain length (b), grain area (c), and TGW (d) for t‐wild‐type and mutant wheat lines. The mean value of each genotype is shown as a red circle. The genotypes of the *TaGW7* homoeologs are shown with lower and upper case letters corresponding to the mutant and wild‐type alleles, respectively, for the A, B, and D genome.

To validate these results, larger populations from the T_3_ generation of YLD2‐5‐1‐41 and YLD2‐5‐1‐52 were evaluated for the kernel size and weight traits. To minimize the possible effects of plant regeneration from the immature embryos on the grain size and weight traits, the phenotypic comparisons between the wild‐type and mutant genotypes were performed only between those T_3_ plants that were derived from the same T_1_ plant. From this point forward, plants with a wild‐type genotype derived from transgenic plants will be referred as ‘t‐wild‐type’. Among the T_3_ plants derived from both YLD2‐5‐1‐41 and YLD2‐5‐1‐52 lines, the grains were significantly wider (1.9–2.2%) in the *TaGW7‐B1* mutants than in the nonmutated T_3_ plants (Tables 1 and 2, Figures 2 and S4). Comparison of grain width between the heterozygous and homozygous *TaGW7‐B1* mutants appears to show the dosage effect of mutations on grain width in the YLD2‐5‐1‐41 family (Table 1, Figure S4). The lack of changes in grain length in the *TaGW7‐B1* mutants suggested that the grain width contributed most to the grain area increase in the YLD2‐5‐1‐41 progeny.

**Table 1:** The TGW and grain morphometric traits in the T_3_ generation plants derived from YLD2‐5‐1‐41

Genotype^a^	*N*	Width (mm)	Length (mm)	Area (mm^2^)	TGW (g)
*AABBDD*	9	3.62 ± 0.033	6.16 ± 0.056	16.09 ± 0.27	40.32 ± 0.88
*AABbDD*	21	3.65 ± 0.031	6.19 ± 0.030	16.37 ± 0.18	41.23 ± 0.76
		0.83%	0.49%	1.74%	2.26%
*AAbbDD*	13	3.7 ± 0.023	6.23 ± 0.038	16.64 ± 0.17	42.31 ± 0.69
		2.21%*	1.14%	3.42%*	4.94%*

a The TGW and grain morphometric measurements for T_3_ plants derived from YLD2‐5‐1‐41 are shown as mean ± standard error. N is number of plants. The phenotype data of each genotype were compared with that of genotype AABBDD using two‐tailed Student's *t*‐test; *significant at 0.01 < *P * < 0.05. The value increase compared with the t‐wild‐type genotype ‘*AABBDD*’ is shown as a percentage.

**Table 2:** The TGW and grain morphometric traits in the T_3_ generation plants derived from YLD2‐5‐1‐52

Genotype	*N*	Width (mm)	Length (mm)	Area (mm^2^)	TGW (g)
*AABBDD*	26	3.67 ± 0.017	6.27 ± 0.033	16.67 ± 0.14	42.72 ± 0.48
*AAbbDD*	7	3.74 ± 0.028	6.29 ± 0.066	16.92 ± 0.24	43.48 ± 1.03
		1.91%*	0.32%	1.50%	1.78%
*AABBdd*	6	3.75 ± 0.032	6.14 ± 0.057	16.73 ± 0.25	43.96 ± 0.72
		2.18%*	−2.07%*	0.36%	2.90%
*AAbbdd*	47	3.76 ± 0.012	6.12 ± 0.020	16.66 ± 0.09	44.07 ± 0.35
		2.45%****	−2.39%****	−0.06%	3.16%*
ANOVA (*P*‐value)		0.00032	1.88e‐05	0.32	0.13

All data are shown as mean ± standard error. N is the number of plants. The phenotype data of each genotype were compared with that of genotype *AABBDD* using the Student's *t*‐test; *significant at 0.01 < *P *<* *0.05, ****significant at *P *<* *0.0001. The value increase relative to the t‐wild‐type genotype ‘*AABBDD*’ is shown as a percentage.

Among YLD2‐5‐1‐52 T_3_ plants, compared with t‐wild‐type, mutations in the *TaGW7‐D1* gene resulted in significantly increased grain width (2.2%) and significantly decreased grain length (2.1%) (Table 2 and Figure 2) without impact on grain area. While statistically nonsignificant, mean TGW was increased in the single genome mutants of both *TaGW7‐B1* and *TaGW7‐D1* compared with t‐wild‐type plants (Table 2). However, statistically significant increases observed for TGW in the *TaGW7‐B1* mutants from YLD2‐5‐1‐41 family suggested that the lack significant TGW change in the YLD2‐5‐1‐52‐derived T_3_ mutants is likely to be associated with insufficient statistical power (Table 1). In the two independent experiments including the YLD2‐5‐1‐52 T_3_ lines, the *TaGW7‐D1* mutants always had wider and shorter grains, and higher TGW compared with the *TaGW7‐B1* mutants, indicating that the *TaGW7* gene mutations in the D genome have stronger phenotypic effects than mutations in the B genome (Table 2, Figures 2 and S3).

**Figure 3:**
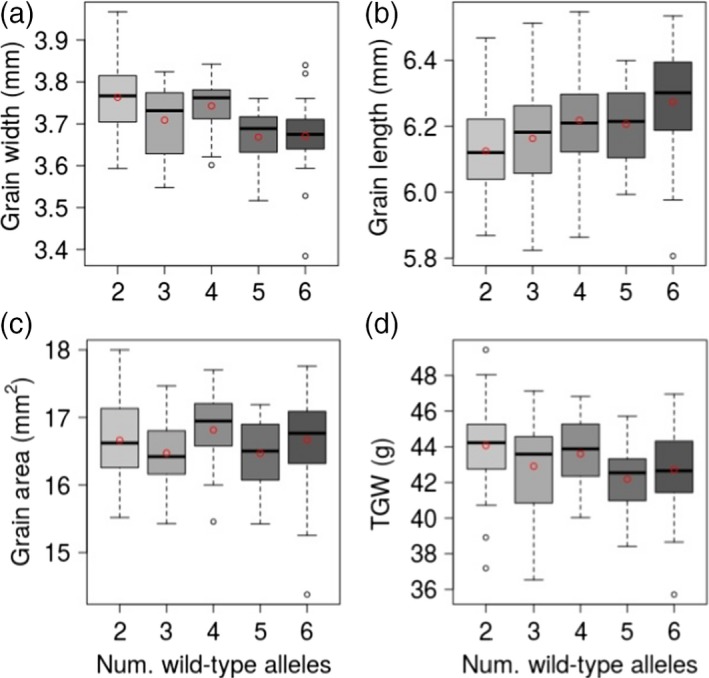
Relationship between the number of the wild‐type (nonmutant) *TaGW7* gene copies and grain morphometric and TGW traits in cv. Bobwhite. Box and whisker plots show the trait distribution for grain width (a), grain length (b), grain area (c), and TGW (d) in the gene‐edited mutant lines grouped based on the number of the functional *TaGW7* gene copies. The mean value of each group is shown as a red circle within the box plot. The number of wild‐type alleles of the *TaGW7* gene is shown on the *x*‐axis.

The dosage‐dependent effects of mutations in *TaGW7‐B1* and *TaGW7‐D1* were supported by a more substantial increase in grain width and decrease in grain length in the double‐genome mutants than in the single genome mutants (Figures 2 and S3, Table 2). The higher TGW in the double‐gene mutants (3.16%) than in the single gene mutants (1.78% and 2.9%) also supported the dosage effect of mutations in the *TaGW7* gene homoeologs (Table 2). To further confirm these results, 116 T_3_ plants were grouped based on the number of nonmutated copies of the *TaGW7* gene. With the increase in the functional *TaGW7* copy number, the grain width and TGW decreased and grain length increased (Figure 3), but change in grain area was not obvious.

### *TaGW7* gene expression pattern in wild‐type and mutant plants

**Figure 4:**
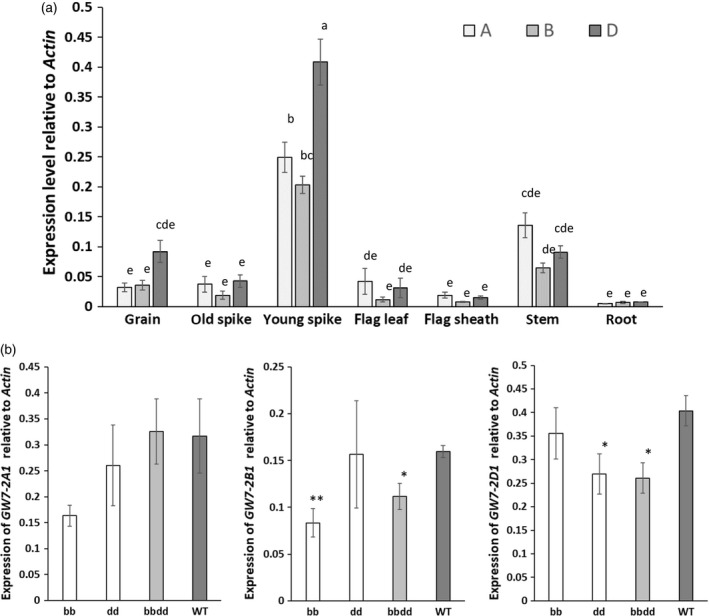
Expression levels of the *TaGW7* gene homoeologs in (a) cv. Bobwhite, and (b) gene‐edited mutants. The quantitative RT‐PCR of the *TaGW7* gene homoeologs was conducted using genome‐specific primers (Figure S1) using *actin* as reference. Expression data for the A, B and D genome gene copies is represented by different shades of gray. Relative expression levels are shown as means ± SEM based on five (a) or three (b) biological replicates. In (a), different letters above the bars indicate significant differences (*P* < 0.05) using Tukey's multiple‐comparison test following one‐way ANOVA. In (b), spikes at the stage when half of the spike emerged from the flag leaf sheath were sampled from the T_3_ generation *TaGW7* mutants or t‐wild‐type plants segregated from the same T_2_ mutant line. The expression levels in mutants was compared with t‐wild‐type plants using *t*‐test; ‘*’ and ‘**’ stand for significance levels of 0.05 and 0.01, respectively.

To better understand the phenotypic effects of the *TaGW7* mutants, the expression levels of *TaGW7* homoeologs in Bobwhite were investigated using quantitative RT‐PCR (Figure S5). The expression of the *TaGW7* homoeologs was substantially higher in nonpollinated young spikes than in other tissues. The D genome copy of the gene expressed at the higher levels than other two homoeologs in nonpollinated young spikes and grain (Figure 4a). These results are consistent with the expression levels of the *TaGW7* homoeologs in cultivars Chinese Spring and Azhurnaya assessed by RNA‐seq analyses (Figure S6) (Ramírez‐González *et al*., 2018). The expression levels of the *TaGW7* homoeologs in the other tissues were lower and did not show significant differences among the genomes. These results suggested the likely association between the higher expression level and the stronger phenotypic effect for the *TaGW7* gene homoeologous copies.

The expression level of *TaGW7* was also evaluated in the plants carrying gene editing mutations in the B and D genomes (Figure 4b). These analyses revealed a substantial reduction of transcripts generated from the edited copies of the *TaGW7* gene, which agrees with *TaGW2* gene expression pattern from our previous study (Wang *et al*., 2018b).

### Gene co‐expression networks associated with *TaGW7*

**Figure 5:**
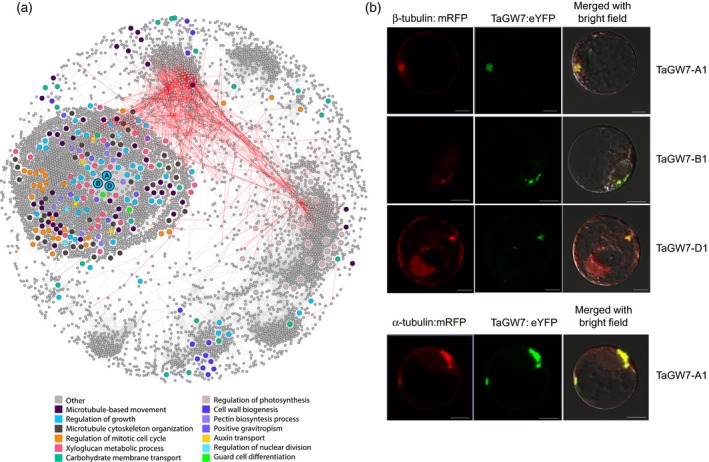
Gene co‐expression network shows association of *TaGW7* with the processes of cell division and morphogenesis. (a) The *TaGW7*‐associated gene co‐expression network. The genes in the network are shown as circles in which colors correspond to different GO terms. Positive and negative PCCs between the gene expression values are shown by gray and red edges, respectively. The three homoeologous copies of the *TaGW7* gene are shown as circles labeled with A, B and D. (b) α‐ and β‐TUBULIN‐6 fused with mRFP protein co‐localize with the TaGW7‐eYFP protein fusions in the wheat protoplasts.

To better characterize the *TaGW7*‐associated biological pathways in wheat, we have constructed *TaGW7*‐centered gene networks (Figure 5a). The expression data from 14 RNA‐seq transcriptome profiling experiments (Ramírez‐González *et al*., 2018), were used to measure pair‐wise expression level correlation between *TaGW7* and other genes in the expression datasets (Table S3). Genes showing a Pearson correlation coefficient (PCC) above 0.6 and in the top 1% of PCC value distribution for all pair‐wise comparisons, were analyzed for functional enrichment in different GO terms. Enriched GO terms include microtubule‐based movement (GO:0007018), regulation of mitotic cell cycle (GO:0007346), regulation of mitotic nuclear division (GO:0007088), auxin transport (GO:0060918), regulation of developmental growth (GO:0040008), and cell wall biogenesis (GO:0042546) (Figure 5a, Tables 3 and S4). Co‐expression of *TaGW7* along with these genes is suggestive of its involvement in the control of cell division and developmentally regulated organ growth in response to auxin signaling. These findings are consistent with the previous studies that showed the association of TONNEAU1‐recruiting motif proteins with protein phosphatase 2A and TONNEAU1 forming the pre‐prophase band (Spinner *et al*., 2013).

**Table 3:** GO enrichment analyses of *TaGW7*‐associated gene network of genes in wheat

GO accession	GO description	Adjusted *P*‐value^a^	Fold change^b^
GO:0007018	Microtubule‐based movement	7.91E‐35	14.9
GO:0060918	Auxin transport	1.26E‐19	26.8
GO:0040008	Regulation of growth	2.40E‐19	20.3
GO:0000226	Microtubule cytoskeleton organization	3.74E‐19	7.4
GO:0010411	Xyloglucan metabolic process	1.65E‐18	16.7
GO:0048638	Regulation of developmental growth	5.44E‐16	20.5
GO:0042546	Cell wall biogenesis	5.20E‐15	10.4
GO:0007346	Regulation of mitotic cell cycle	1.68E‐14	6.8
GO:0010109	Regulation of photosynthesis	1.94E‐11	13.2
GO:0045489	Pectin biosynthetic process	4.81E‐11	28.0
GO:0051783	Regulation of nuclear division	5.10E‐11	11.0
GO:0010052	Guard cell differentiation	3.58E‐10	230.7
GO:0009958	Positive gravitropism	1.69E‐09	24.3
GO:0034219	Carbohydrate transmembrane transport	2.27E‐09	5.0

a Fisher's exact test *P*‐values were adjusted using the Benjamini−Hochberg correction.

b Fold change in the representation of genes from a given GO term in the *TaGW7*‐associated network compared with the genome‐wide estimates.

The validity of *TaGW7*‐associated co‐expression network is corroborated by the substantial overlap of the networked genes with the protein−protein interacting partners identified for OsGW7 in rice (Wang *et al*., 2015a). Out of 18 rice proteins that were shown to interact with OsGW7 in yeast two‐hybrid assay, seven had wheat orthologs that were part of the *TaGW7*‐associated network (Table S5).

To further investigate the involvement of *TaGW7* into the control of cell division, we determined its cellular localization relative to α‐ and β‐tubulin, the building blocks of microtubule arrays (Hashimoto, 2015). We found that the *TaGW7* gene co‐expresses with the β‐tubulin gene (PCC ≥ 0.6) (Figure 5b, Table S3). The  TaGW7‐A1, TaGW7‐B1, and TaGW7‐D1 proteins fused with yellow fluorescent protein (eYFP) (Figure S7), were transiently co‐expressed in the wheat protoplasts along with *α‐* and *β‐tubulin* genes fused with mRFP (Figure 5b). The fluorescence signals from the TaGW7 fusion proteins co‐localized with α‐ and β‐tubulin‐eYFP signals suggest that these proteins occupy the same cellular compartments (Figure 5b).

### Evolutionary history of the *TaGW7* genes in wheat

**Figure 6:**
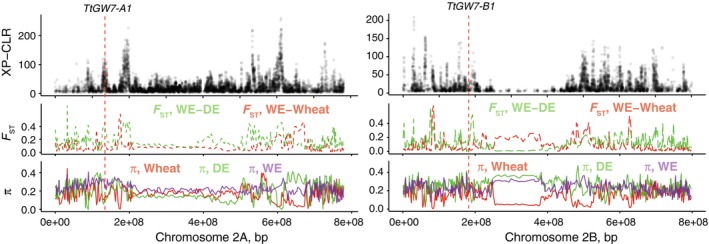
Patterns of genetic diversity and differentiation on chromosomes 2A and 2B. Selective sweeps on chromosomes 2A and 2B associated with wheat domestication were identified using the XP‐CLR scans performed using wild emmer (WE) as the reference population and domesticated emmer (DE) as the target population. Average pair‐wise genetic differentiation (*F*_ST_) and average pair‐wise diversity per SNP site (π) in the corresponding regions in the populations of wheat, WE and DE was estimated using SNPs in 2‐Mb sliding windows. *F*_ST_ was measured between wild and domesticated emmer (*F*_ST (WE‐DE)_) and between WE and wheat (*F*_ST (WE‐Wheat)_). The positions of the *OsGW7* gene orthologs are shown by the vertical dashed red lines.

To better understand the evolution of the *TaGW7* gene locus, we performed the analyses of SNP diversity in a set of 818 hexaploid and tetraploid wheat accessions including 34 accessions of wild emmer, 40 accessions of domesticated emmer, 286 landraces and 458 cultivars of hexaploid wheat (He *et al*., 2019; Avni *et al*., 2017). Population differentiation XP‐CLR test (Chen *et al*., 2010) applied to tetraploid wild and domesticated emmer wheat showed that the *TtGW7‐A1* gene, the ortholog of *OsGW7* in wild emmer, is located in the middle of domestication selective sweep region on chromosome 2A (Figure 6). This region also showed the reduction of genetic diversity (π) in domesticated emmer and bread wheat and increased genetic differentiation (*F*_ST_) between wild and domesticated wheat (Figure 6). These results are consistent with strong selection at the *TtGW7‐A1* gene region during domestication. While we did observe some increase in the level of *F*_ST_ between wild and domesticated emmer near the *TtGW7‐B1* gene on homoeologous chromosome 2B, this genic region did not show elevated XP‐CLR values consistent with strong selective sweep (Figure 6).

Using the same exome capture data, we further evaluated the haplotypic diversity of the *TaGW7* genes in the populations of hexaploid wheat landraces and cultivars, and tetraploid wild and domesticated emmer (He *et al*., 2019). We have extracted SNPs from the genic regions (intron+exon) of the *TaGW7* gene homoeologs in the A, B, and D genomes (Table S6). In the A genome of hexaploid wheat, we have identified five haplotypes (Ha1–Ha5) of the *TaGW7* gene (Table 4, Table S6).

**Table 4:** Haplotypic diversity of the *TaGW7* gene homoeologs in hexaploid and tetraploid wheat

Gene in wheat	Species	Improvement status	Number of accessions	Haplotype	Frequency
*TaGW7*‐*A1*	*T. aestivum* ssp. *aestivum*	Cultivars	306	H1a	0.843
			39	H2a	0.107
			17	H3a	0.047
			1	H4a	0.003
		Landraces	210	H1a	0.850
			3	H2a	0.012
			31	H3a	0.126
			3	H5a	0.012
	*T. turgidum* ssp. *dicoccon*	Domest.	28	H1a	1.000
	*T. turgidum* ssp. *dicoccoides*	Wild	6	H1a	0.194
			14	H5a	0.452
			11	H6a	0.355
*TaGW7‐B1*	*T. aestivum* ssp. *aestivum*	Cultivars	203	H1b	0.573
			136	H2b	0.384
			4	H3b	0.011
			11	H4b	0.031
		Landraces	163	H1b	0.709
			61	H2b	0.265
			6	H3b	0.026
	*T. turgidum* ssp. *dicoccon*	Domest.	14	H1b	0.700
			6	H2b	0.300
	*T. turgidum* ssp. *dicoccoides*	Wild	4	H1b	0.133
			26	H2b	0.867
*TaGW7‐D1*	*T. aestivum* ssp. *aestivum*	Cultivars	341	H1d	0.991
			3	H2d	0.009
		Landraces	242	H1d	0.992
			2	H2d	0.008

Both cultivars Bobwhite and Chinese Spring had haplotype H1a. The analysis of hexaploid wheat showed that H1a is the most abundant haplotype present at the frequency of 84.3% and 85.0% in cultivars and landraces, respectively. The frequency of remaining haplotypes was different between wheat landraces and cultivars. Compared with landraces, the H2a haplotype in cultivars increased in frequency from 1.2% to 10.7%, whereas haplotype H3a decreased in frequency from 12.6% to 4.7%. Haplotype H4a was not identified in landraces, whereas haplotype H5a was present at a frequency of 1.2% in landraces, but was not detected in cultivars. The analysis of domesticated emmer wheat population, the direct ancestor of the hexaploid wheat's A and B genomes (Avni *et al*., 2017; He *et al*., 2019), also identified only one haplotype, H1a. In the wild emmer wheat population, haplotype H1a was present at significantly lower frequency (19.4%) than in domesticated emmer (Fisher's exact two‐tailed *P‐*value <0.0001). The most common haplotype H5a (45.2%) from wild emmer was not detected in the analyzed sample of domesticated emmer, but was found at low frequency (1.2%) in wheat landraces (Table 4). These findings suggested that the H1a haplotype was one of the likely targets of domestication selection and was inherited by bread wheat from domesticated emmer.

The analysis of the *TaGW7‐B1* gene diversity in cultivated bread wheat identified four haplotypes H1b, H2b, H3b, and H4b with the frequencies of 57.3%, 38.4%, 1.1% and 3.1%, respectively (Tables 4 and S6). The reference cultivar Chinese Spring and cultivar Bobwhite had common haplotypes H1b and H2b, respectively. In the population of landraces, the H4b haplotype was missing, and H1b, H2b, and H3b were present at the frequencies of 70.9%, 26.5%, and 2.6%, respectively. Even though both common H1b and H2b haplotypes were detected in the wild and domesticated emmer wheat (Table 4), we observed significant differences in their frequency (Fisher's exact two‐tailed *P‐*value <0.0001). The H1b haplotype found in 13.3% of wild emmer accessions was detected in nearly 70% of domesticated emmer accessions, suggesting that H1b was the possible target of domestication selection. The fact that both H1b and H2b haplotypes are found in both wild and domesticated emmer populations might be one of the reasons why XP‐CLR and F_ST_ tests did not show the evidence of substantial allele frequency differentiation in the *TtGW7‐B1* region (Figure 6 and Table S6).

Out of two haplotypes identified for the *TaGW7‐D1* gene, the H1d haplotype was nearly fixed and found in 99.1% of accessions, including cv. Chinese Spring and cv. Bobwhite (Table 4, Table S6). This major haplotype was identical to the one in the reference genome of *Ae. tauschii*, the diploid wild relative of the D genome (Luo *et al*., 2017).

## Discussion

Early comparative analyses of the related plant species revealed parallelism in the variation of their morphological and physiological traits (Vavilov, 1922). Numerous genetic studies have shown that many of these traits have a common genetic basis. With the high‐density genetic maps and reference genome sequences available for multiple crops, this strategy of using comparative genomics for understanding the genetic basis of trait control in related crops appeared to be effective and allowed to reduce time and efforts needed to identify causal genes. During the last several years, several genes controlling grain size and weight identified in rice were shown to underlie similar traits in wheat (Zhang *et al*., 2012a; Dong *et al*., 2014; Wang *et al*., 2018b; Zhang *et al*., 2019). Here, we characterized the wheat *TaGW7* gene, the ortholog of the *OsGW7* gene in rice. Its downregulation in rice correlated with the production of shorter and wider grains (Wang *et al*., 2015a). Our study demonstrated that the gene‐edited alleles of *TaGW7* showed phenotypic effects similar to that observed in rice, and produce wider and shorter wheat grain. However, mutants with two copies of edited *TaGW7* in wheat increased TGW (~3.2%), which was not observed in a rice line with the down‐regulated *OsGW7* (Wang *et al*., 2015a). It is possible that even though the genetic architecture of grain dimension traits in wheat and rice tends to be conserved, the phenotypic effects of gene perturbations in related species can sometimes be different due to interactions with the divergent species‐specific pathways.

Variation among genes in the relative contribution of individual homoeologs to the total expression level of a homoeologous gene set is a common phenomenon in wheat (Akhunova *et al*., 2010; Ramírez‐González *et al*., 2018). However, until recently the role of this factor in the contribution of gene homoelogs to trait variation received little attention. Our earlier study showed that the effect sizes of mutations in the individual *TaGW2* gene homoeologs correlated well with their levels of expression relative to other gene copies (Wang *et al*., 2018b). While we did not investigate the phenotypic effects of mutations in the *TaGW7‐A1* gene, it appears that *TaGW7*, similar to *TaGW2*, also showed similar relationships between the effect sizes of mutations and the expression levels of individual homoeologs. Interestingly, in both genes, the edited copy in the D genome of cultivar Bobwhite contributed the most to trait variation and gene expression, indicating that the merger with the D genome ancestor during bread wheat origin could have played important roles in the evolution of yield component traits. In addition to homoeolog‐specific effects of mutations, the gene editing of both *TaGW2* (Wang *et al*., 2018b) and *TaGW7* revealed dosage‐dependent effects on grain morphometric and weight traits. Responsiveness of the *TaGW7‐*connected developmental pathway to gene dosage variation is also confirmed in rice, in which the number of the *OsGW7* gene copies had a strong effect on grain shape (Wang *et al*., 2015b). Our observations are consistent with the hypothesis suggesting that many duplicated homoeologs in allopolyploid wheat retain their functionality and may impose dosage‐mediated effects on trait variation (Akhunova *et al*., 2010; Díaz *et al*., 2012; Krasileva *et al*., 2017; He *et al*., 2019).

The involvement of microtubules in embryo and endosperm morphogenesis has been previously demonstrated (Brown *et al*., 1994; Pignocchi *et al*., 2009; Hashimoto, 2015; Luptovčiak *et al*., 2017). Microtubule topology during the three major stages of cereal endosperm development (syncytial, cellularization and differentiation) showed close relationships with the cell shape, cell wall development and positioning of cell organelles (Brown *et al*., 1994). Consistent with these observations, the enrichment analysis of the *TaGW7*‐centered networks showed significant over‐representation of genes involved in biological processes connected with microtubule‐based movement (GO:0007018), regulation of mitotic cell cycle (GO:0007346), regulation of mitotic nuclear division (GO:0007088) and regulation of developmental growth (GO:0040008), and cell wall biogenesis (GO:0042546). Recent findings suggest that the ability of microtubule arrays to interact with multiple proteins plays a critical role in the regulation of these biological processes linked with organ morphogenesis. The *OsGW7* and *TaGW7* genes are homologs of *TRM1* that are likely to affect grain dimension traits by influencing cell elongation and division (Wang *et al*., 2015a). The TRM1 protein was shown to interact with TON1 (Drevensek *et al*., 2012), which in turn interacts with microtubule arrays involved in the formation of pre‐prophase band (Hashimoto, 2015). An interaction between rice homologs of TRM1 and TON1 was confirmed using the yeast two‐hybrid assay (Wang *et al*., 2015a). Another interacting partner of TRM protein in both rice and *Arabidopsis* is a protein phosphatase 2A (PP2A) encoded by the *FASS/TON2* gene (Drevensek *et al*., 2012; Wang *et al*., 2015a), which together with TRM and TON1 was shown to form PPB and be involved in the spatial control of cell division in *Arabidopsis* (Spinner *et al*., 2013). Our finding that *TaGW7* co‐localizes with α‐ and β‐tubulins, the main building blocks of microtubule arrays, is consistent with the co‐localization and interaction studies in *Arabidopsis* demonstrating that TRM1 can be recruited to microtubule arrays through a positively charged basic domain, one of the main features of the TRM proteins (Drevensek *et al*., 2012). These results suggested that protein−protein interactions including microtubule arrays and TRM are important part of the pathways that control grain or fruit dimension traits in wheat and other crops. Consistent with this conclusion is the finding that, in rice, a mutation in an *α‐tubulin* gene (*Srs5* mutant) affected grain dimension traits (Segami *et al*., 2012). Similarly, the interaction between the OVATE family proteins and TRM were shown to regulate cell division and alter fruit shape in tomato (Wu *et al*., 2018).

Wheat domestication was accompanied by an increase in kernel size and weight (Peleg *et al*., 2011). The analyses of selective sweeps, genetic differentiation and haplotypic diversity suggested that the *TaGW7* gene was one of the likely targets of domestication selection. The shorter and wider grain characteristics of the domesticated forms of tetraploid wheat compared with its wild tetraploid ancestor (Nave *et al*., 2016) could have resulted from selection acting on grain dimension traits modulated by *TaGW7*. Strong selection on the H1a haplotype of the *TaGW7‐A1* gene was consistent with its relatively low frequency in wild emmer (19.4%), fixation in domesticated emmer and high frequency in wheat cultivars (84.3%) and landraces (85%). At the same time, the most frequent haplotype H5a from wild emmer was not detected in domesticated emmer and wheat cultivars, and present at low frequency (1.2%) in wheat landraces. The latter was consistent with the evidence of higher rates of gene flow from wild emmer into wheat landraces than into cultivars (He *et al*., 2019). Similar changes in the haplotype frequency accompanying wheat domestication and improvement were found for the *TaGW7‐B1* gene on chromosome 2B. Our data suggested that only one of the *TaGW7* haplotypes or its close derivatives reached high frequency in domesticated and cultivated wheats. This observation can potentially explain the lack of overlap between the *TaGW7* gene and previously identified QTL for yield component traits (Rasheed *et al*., 2014; Zanke *et al*., 2015; Yan *et al*., 2019). It is possible that wheat populations have been nearly fixed for the beneficial alleles of *TaGW7* precluding the identification of their phenotypic effects using genome‐wide association mapping. This finding is consistent with studies in rice demonstrating that some alleles of yield component genes with strong effect are rare in population (Zhang *et al*., 2012b; Hu *et al*., 2015; Song *et al*., 2015).

The phenotypic effects of genes that lost diversity during domestication and selection are not easily detectable in genetic mapping studies based on the analyses of only domesticated forms of crops. Our results suggested that the CRISPR‐Cas9‐based editing of the wheat orthologs of genes with the known phenotypic effects in other crops can help to overcome some of these limitations and select targets for engineering new variants of genes with beneficial effect on agronomic traits broadening genetic diversity available for wheat improvement.

## Experimental Procedures

### Analysis of the *TaGW7* gene sequences

The coding sequence (CDS) of the *OsGW7* gene from rice (Wang *et al*., 2015a) was used to perform BLASTN search using the genomes of allohexaploid wheat *Triticum aestivum* ssp. *aestivum* (genome formula AABBDD) (The International Wheat Genome Sequencing Consortium (IWGSC), 2018), and its tetraploid ancestor *T. turgidum* ssp. *dicoccoides* or wild emmer (genome formula AABB) (Avni *et al*., 2017) and diploid ancestor *Aegilops tauschii* (genome formula DD) (Luo *et al*., 2017) from the Ensembl Plants database (plants.ensembl.org). The orthologous genes were identified based on the BLASTN hits showing sequence identity higher than 70%, e‐value less than 1e^−5^ and located on the wheat chromosomes 2A, 2B, and 2D, which are syntenic to rice chromosome 7 (Sorrells *et al*., 2003). Applying these sequence similarity thresholds revealed no hits on other chromosomes were detected.

The gene sequences including both introns and exons were used to build the phylogenetic tree. Alignment was performed using MUSCLE (Edgar, 2004). Pair‐wise distances between the aligned gene sequences after removal of regions carrying insertions and deletions were calculated using the Kimura‐2‐parameter model implemented in program MEGA v.6.06. The gene tree was constructed using the Neighbor‐Joining method followed by bootstrap testing with 1000 replications. The cDNA was prepared from the total RNA isolated from the spikes of cv. Bobwhite before anthesis. The CDS of the *TaGW7* gene in cv. Bobwhite was amplified using the genome‐specific primers designed to the 5′ and 3′ UTRs of the *TaGW7* gene homoeologs (Table S1). PCR was performed using NEBNext Ultra II Q5^®^ Master Mix (New England Biolabs, Catalog #M0544S, Ipswich, MA, USA) following the manufacturer's protocol. The PCR amplicons were subcloned into pCR‐Blunt vector using the Zero Blunt^™^ PCR Cloning Kit (ThermoFisher Scientific, Waltham, MA, USA, Catalog #K270020). Plasmid DNA was extracted from three to five bacterial colonies for each PCR product using QIAprep Spin Miniprep Kit (QIAGEN, Catalog #27106, Hilden, Germany). The sequence of each clone was determined using the Sanger method and used to infer the consensus sequence of each gene.

### Design and validation of CRISPR‐Cas9 targets in the *TaGW7* gene

The CIRSPR‐Cas9 targets within the *TaGW7* gene were designed as described previously (Wang *et al*., 2016, 2018a). Briefly, the CDS of *TaGW7‐A1* was analyzed using sgRNAscorer 1.0 (http://crispr.med.harvard.edu/sgRNAScorer). The highest scoring gRNA targets were compared with the wheat reference genome RefSeq v1.0 (The IWGSC, 2018) using the BLASTN program. The gRNA target sequences conserved between at least two wheat genomes and not showing similarity outside of the target region were used to create the gRNA constructs.

Each gRNA was synthesized as two complementary oligonucleotides with four‐nucleotide overhangs at both termini (Table S1). The oligonucleotides were annealed and subcloned into CRISPR‐Cas9 plasmid pA9Cas9sg as described (Wang *et al*., 2018a). The genome editing efficiency of each gRNA construct was tested by transient expression in the wheat protoplasts (Table S2), and the NGS of gRNA target region (Wang *et al*., 2016).

### Regeneration of transgenic plants and genotyping of the *TaGW7* gene mutants

The pA9Cas9sg constructs targeting two sites, referred as GW7T2 and GW7T6, were mixed in equimolar amounts with the *bar*‐gene carrying pAHC20 construct and biolistically transformed into the embryos of wheat cultivar Bobwhite. The T_0_ plants were regenerated as described in our previous study (Wang *et al*., 2018b). Three primer pairs spanning the CRISPR‐Cas9 construct were used to identify CRISPR‐Cas9 positive plants using PCR (Table S1). The target sites of the CRISPR‐Cas9 positive T_0_ plants and plants from the T_1_, T_2_ and T_3_ generations were analyzed by NGS of pooled barcoded PCR amplicons, as described previously (Wang *et al*., 2018a). The primers used for NGS are listed in Table S1.

### Plant growth conditions

The plants from the T_3_ generation of YLD2‐5‐1‐52 line were grown in a growth chamber under 16 h light/8 h dark with day and night temperature set to 24°C and 21°C, respectively. All plants in other experiments were grown in a greenhouse under 16 h light/8 h dark treatments at 21–24°C. The T_1_ generation plants were grown in the 0.2 L square pots filled with SunGro soil (Sun Gro Horticulture, Agawam, MA, USA). All other plants were grown in 1 L square pots filled with three parts of soil mix (volume ratio was 20 soil:20 peat moss:10 perlites:1 CaSO_4_) and topped with one part of the SunGro soil mix (Sun Gro Horticulture, Agawam, MA, USA). Plants in the greenhouse were arranged according to the complete randomized design.

### The expression analysis of *TaGW7* in cv. Bobwhite and its mutants

To analyze the *TaGW7* gene expression in cv. Bobwhite, total RNA was isolated from the plants at anthesis stage from the following tissues on the main spike tiller: 1‐week‐old grain, spike without the grain, flag leaf, flag leaf sheath, and stem. In addition, one nonpollinated spike on another tiller and roots were sampled from the same plants. RNA was extracted using TRIzol (Thermo Fisher Scientific, catalog #15596018) following the manufacture's protocol. The first‐strand cDNA was synthesized using ‘SuperScript™ III First‐Strand Synthesis SuperMix’ (Thermo Fisher Scientific, Catalog #11752‐250) according to Thermo Fisher's recommendations. The *TaGW7‐A1*,* ‐B1* and *‐D1* homoeolog‐specific RT‐PCR primers (Table S1) were designed and validated by PCR using the DNA isolated from the Chinese Spring nulli‐tetrasomic lines (Figure S5). Quantitative RT‐PCR was performed using the iQ^™^ SYBR Green Supermix (Bio‐Rad, Catalog #170‐8882, Hercules, CA, USA) following the manufacturer's instructions with the *actin* gene as reference.

To investigate the possible effects of mutations in one or two copies of the *TaGW7* gene on the expression of nonmutated gene copies, the relative expression levels of each gene copy in the single‐ and double‐gene mutant lines were characterized. For this purpose, we used total RNA isolated from the spikes at the stage when half of the spike emerged from the flag leaf sheath. At least three biological replicates were used for expression analyses.

### Intracellular localization of TaGW7

To study the cellular localization of TaGW7, the CDS of each gene homoeolog was fused to the 3′‐ ends of the *eYFP* gene from plasmid pA9ReYFP (Figure S7). The CDS was amplified using primers GW7AD/B_CDS_F‐9RY and GW7_CDS_R‐9RY (Table S1) and inserted into  pA9ReYFP between the *Kpn*I and *Mlu*I cut sites. The vector constructs were validated by Sanger sequencing.

The constructs expressing microtubule‐localized mRFP were created by fusing α‐ and β‐TUBULIN‐6 with the C‐terminus of mRFP in plasmid pA9mRFP (Wang *et al*., 2018a). For this purpose, the coding regions of the *α*‐ and *β*‐*TUBULIN‐6* gene orthologs (TraesCS4A02G257800 and TraesCS1B02G269400) were amplified using PCR from the cDNA prepared using RNA isolated from the Bobwhite spikes. The 18 bp‐long glycine−serine linker was inserted between *α*‐ and *β*‐*TUBULIN‐6* and *mRFP* by adding tails to the 5′‐ends of PCR primers. The PCR products generated using NEBNext Ultra II Q5^®^ Master Mix (New England Biolabs, Catalog #M0544S) were subcloned into pA9mRFP plasmid using NEBuilder^®^ HiFi DNA Assembly Cloning Kit (New England Biolabs, Catalog #E5520S). These constructs, from this point forward, will be referred to as pA9mRFP‐*α*‐*TUBULIN‐6* and pA9mRFP‐*β*‐*TUBULIN‐6*. The constructs expressing the *TaGW7*‐eYFP fusion proteins were co‐transformed into Bobwhite protoplasts along with pA9mRFP‐*α*‐*TUBULIN‐6* and pA9mRFP‐*β*‐*TUBULIN‐6*. After 18 h of incubation at room temperature, the fluorescent signals were analyzed using the LSM 700 Confocal Laser Scanning Microscope (Zeiss Inc, Jena, Germany).

### Collection of grain shape, size and thousand grain weight data

The three main spikes were collected from each plant. The grain width, grain length, grain area and TGW were measured separately for each spike, and used to calculate the mean values for each plant. Information about the sample sizes for each phenotyping experiment is provided in the respective sections in the Results. Trait measurements were conducted using a MARVIN seed analyzer (GTA Sensorik GmbH, Germany), as described previously (Wang *et al*., 2018b).

### Co‐expression analysis of the *TaGW7* gene

The expression data (transcripts per million, TPM) of 209 RNA‐seq samples from the developmental time‐course of Azhurnaya were downloaded from the Wheat Expression Browser (http://www.wheat-expression.com/download) (Ramírez‐González *et al*., 2018). We used gene expression levels estimated from the sum of all isoforms. A quantile robust normalization was performed followed by the log_2_ transformation of the TPM values. Our co‐expression network was comprised of two parts: (i) a global network based on top 10 000 genes that had the highest levels of gene expression variability assessed based on the standard deviation; and (ii) a local network build using the top 1% genes that showed the strongest correlation with the levels of the *TaGW7* gene expression. We used Gephi (https://gephi.org) to visualize the network. The gene pairs with the PCC less than −0.90 or larger than 0.98 in the global network were visualized. The gene pairs with absolute PCC larger than 0.60 in the local network were visualized along with the global network.

### GO enrichment of *TaGW7* co‐expressed neighbors

In addition to the Gene Ontology data released by the IWGSC, we used BLAST2GO to generate more detailed functional annotations for both high‐quality (HC) and low‐quality (LC) gene models reported for the wheat genome assembly RefSeq v.1.0 (The IWGSC, 2018). All protein sequences were compared with the NCBI nonredundant sequence database using the BLAST tool. The BLAST output was processed using blast2go (blast2go_cli_v1.3.3) to extract GO terms for each gene. Fisher's exact test was used to determine the significance of the enrichment for every GO term, followed by Benjamini−Hochberg correction (Benjamini and Hochberg, 1995).

### Genetic diversity analyses

We used XP‐CLR statistic (Chen *et al*., 2010) to identify the genomic regions showing the signatures of selective sweeps associated with wheat domestication. For this purpose, we used exome capture data generated for the accessions of tetraploid wild and domesticated emmer wheat (Avni *et al*., 2017). In the XP‐CLR scan, 34 accessions of wild emmer were used as a reference population and 40 accessions of domesticated emmer were used as a test population. The XP‐CLR scan was run with the grid size of 50 kb, the window size of 1 cM, the maximum number of SNP within a window set to 200 and the inter‐variant linkage disequilibrium (*r* ^2^) less than 0.95.

The genetic diversity analyses of the *TaGW7* gene in the populations of wheat 286 landraces and 458 cultivars were performed using previously described whole exome capture SNP data collected for a geographically and genetically diverse panel of accessions (He *et al*., 2019). The SNP variants were extracted from the *TaGW7* gene regions in the A, B, and D genomes of wheat (http://wheatgenomics.plantpath.ksu.edu/1000EC).

### Statistical analysis of data

The distribution of grain length, grain width, grain area and TGW data was visualized using the box and whisker plots. One‐way ANOVA was applied to compare the significance of inter‐group differences followed by post‐hoc Tukey's test. The Student's *t*‐test was applied to assess the significance of difference between the two groups of data.

## Availability of Data and Material

CRISPR‐Cas9 gene editing constructs and wheat lines are available upon request.

## Materials Requests

Materials requests should be addressed to Eduard Akhunov (eakhunov@ksu.edu).

## Author Contributions

WW conducted gene editing experiments, generated and analyzed gene expression data, drafted the manuscript; QP analyzed gene editing events using NGS and conducted gene expression analysis; BT conducted plant transformation experiments; FH constructed gene expression networks and performed diversity data analyses; YC performed biolistic transformation of wheat embryos; AA designed experiments for NGS analysis of editing events and performed NGS; GB validated constructs by Sanger sequencing; HT performed biolistic transformation of wheat embryos with the gene editing constructs and coordinated project; EA conceived idea, designed gene editing experiments, coordinated project, analyzed data and wrote the manuscript.

## Conflict of Interest

The authors declare that they have no conflict of interests.

## Supporting information


**Figure S1.** The CDS and predicted protein sequence of the *TaGW7* gene in cultivars Chinese Spring and Bobwhite.
**Figure S2.** Phenotypic effects of mutations induced by CRISPR‐Cas9 in the *TaGW7* gene in T_2_ generation plants.
**Figure S3.** Phenotypic effects of mutations induced by CRISPR‐Cas9 in the *TaGW7* gene in the T_3_ generation plants derived from YLD2‐5‐1‐52.
**Figure S4.** Phenotypic effects of mutations induced by CRISPR‐Cas9 in the *TaGW7* gene in the T_3_ generation plants derived from YLD2‐5‐1‐41.
**Figure S5.** Validation of the *TaGW7* gene homoeolog‐specific primers for RT‐PCR.
**Figure S6.** The expression levels of the *TaGW7* homoeologs in cv. Azhurnaya and cv. Chinese Spring.
**Figure S7.** The sequences of pA9ReYFP constructs.

**Table S1.** List of primers and oligonucleotides used in the study.

**Table S2.** Assessment of gRNA editing efficiency in the wheat protoplasts.

**Table S3.** List of genes co‐expressed with the *TaGW7* gene homoeologs.

**Table S4.** GO terms significantly enriched in the *TaGW7*‐associated gene networks.

**Table S5.** Wheat genes orthologous to genes in rice that encode proteins interacting with rice OsGW7 in the yeast two‐hybrid (Y2H) assay.
**Table S6.** Haplotypes of the *TaGW7* genes identified in hexaploid and tetraploid wheat.
