# Supplementary material for: Gene editing of the wheat homologs of TONNEAU1‐recruiting motif encoding gene affects grain shape and weight in wheat
Source: Plant J. 2019 Jul 28;100(2):251–64. doi: 10.1111/tpj.14440 (PMC6851855; doi:10.1111/tpj.14440)
Supplement: Supplementary file 6 — Table S5. Wheat genes orthologous to genes in rice that encode proteins interacting with rice OsGW7 in the yeast two‐hybrid (Y2H) assay. Table S6. Haplotypes of the TaGW7 genes identified in hexaploid and tetraploid wheat. [file TPJ-100-251-s006.docx]

**Supplementary Table 5.** Wheat genes orthologous to genes in rice that encode proteins interacting with rice GW7 in the yeast two-hybrid (Y2H) assay.

| Rice genes IDs | GW7 interactors in Y2H screen(Wang et al., 2015*) | Wheat gene orthologs co-expressed with TaGW7 |
| --- | --- | --- |
| LOC_Os01g06060 | Gibberellin receptor GID1L2 |  |
| LOC_Os01g56200 | NPR1-like protein |  |
| LOC_Os01g62760 | Protein phosphatase 2C |  |
| LOC_Os01g74610 | Zinc finger, C3HC4 type domain containing protein | TraesCS5D02G509700 |
| LOC_Os02g32030 | Elongation factor | TraesCS5D02G509700 |
| LOC_Os02g45130 | Protein kinase |  |
| LOC_Os02g57280 | CBS domain containing membrane protein | TraesCS6A02G392100, TraesCS6B02G432300, TraesCS6D02G378000 |
| LOC_Os03g02270 | Tetratricopeptide repeat (TPR)-like superfamily protein | TraesCS5A02G489100 |
| LOC_Os03g03910 | Catalase domain containing protein | TraesCS4D02G322700, TraesCS5A02G498000 |
| LOC_Os03g04310 | bHLH transcription factor |  |
| LOC_Os03g08010 | Elongation factor Tu |  |
| LOC_Os05g05710 | FASS/PP2A |  |
| LOC_Os05g41070 | bZIP transcription factor |  |
| LOC_Os07g33850 | Ras-related small GTP-binding protein |  |
| LOC_Os11g01170 | TON1b |  |
| LOC_Os11g03390 | SMAD/FHA domain containing protein |  |
| LOC_Os11g06020 | Homeobox domain containing protein | TraesCS4B01G114600, TraesCS4A01G200800 |
| LOC_Os11g47970 | AAA-type ATPase family protein | TraesCS4A02G177500, TraesCS4B02G140300, TraesCS4D02G135000 |

*Wang, S., S. Li, Q. Liu, K. Wu, J. Zhang et al., 2015 The OsSPL16-GW7 regulatory module determines grain shape and simultaneously improves rice yield and grain quality. Nat. Genet. 47: 1–7.

**Supplementary Table 6.** Haplotypes of the *TaGW7* genes identified in hexaploid and tetraploid wheat.

| **TaGW7-A1 gene** | | | | | | | | | |
| --- | --- | --- | --- | --- | --- | --- | --- | --- | --- |
| **Chr** | **SNP coordinates in RefSeq v1.0** | **Ref. allele** | **Alt. allele** | **Haplotypes** | | | | | |
|  |  |  |  | **H1a** | **H2a** | **H3a** | **H4a** | **H5a** | **H6a** |
| 2A | 135191850 | A | C | A | A | C | A | C | C |
| 2A | 135193968 | C | T | C | C | C | T | C | C |
| 2A | 135194439 | A | G | A | A | G | A | A | A |
| 2A | 135195006 | G | A | G | A | G | G | G | G |
| 2A | 135195072 | C | T | C | C | C | T | C | T |
| 2A | 135196720 | A | G | A | A | A | G | A | A |
|  |  |  |  |  |  |  |  |  |  |
| **TaGW7-B1 gene** | | | | | | | | | |
| **Chr** | **SNP coordinates in IWGSC RefSeq v1.0** | **Reference allele** | **Alternative allele** | **Haplotypes** | | | |  |  |
|  |  |  |  | **H1b** | **H2b** | **H3b** | **H4b** |  |  |
| 2B | 182012425 | G | A | G | G | G | A |  |  |
| 2B | 182013653 | G | A | G | A | G | G |  |  |
| 2B | 182014205 | C | T | C | C | T | C |  |  |
|  |  |  |  |  |  |  |  |  |  |
| **TaGW7-D1 gene** | | | | | | | | | |
| **Chr** | **SNP coordinates in IWGSC RefSeq v1.0** | **Reference allele** | **Alternative allele** | **Haplotypes** | |  |  |  |  |
|  |  |  |  | **H1d** | **H2d** |  |  |  |  |
| 2D | 128396800 | C | T | C | T |  |  |  |  |
| 2D | 128397122 | C | T | C | C |  |  |  |  |
| 2D | 128399341 | T | G | T | T |  |  |  |  |
| 2D | 128399673 | C | T | C | C |  |  |  |  |
