# Supplementary material for: Gene editing of the wheat homologs of TONNEAU1‐recruiting motif encoding gene affects grain shape and weight in wheat
Source: Plant J. 2019 Jul 28;100(2):251–64. doi: 10.1111/tpj.14440 (PMC6851855; doi:10.1111/tpj.14440)
Supplement: Supplementary file 7 [file TPJ-100-251-s007.docx]

**Supplementary Figure 1.** The CDS and predicted protein sequence of the *TaGW7* gene in cultivars Chinese Spring and Bobwhite. The CDS sequences of the *TaGW7* genes are downloaded from the Ensembl Plants (<https://plants.ensembl.org/index.html>). The homoeologs of the *TaGW7* gene on chromosomes 2A, 2B and 2D are TraesCS2A02G176000, TraesCS2B02G202300, and TraesCS2D02G183400, respectively. The start and stop codons are highlighted with yellow color. The boundaries of different exons are marked with grey or dark grey colors. The SNP and amino acid variations between Chinese Spring and Bobwhite sequences are shown in brackets and highlighted with red color.

**Supplementary Figure 2. Phenotypic effects of mutations induced by CRISPR-Cas9 in the *TaGW7* gene in T2 generation plants.** The phenotypic data are shown as means ± SE; the number of analyzed plants are 4, 6, 6, 3 for genotypes *AABbdd, AABbDD,* *AAbbDD* and *AABBDD,* respectively. The intergroup means for genotypes *AABbdd* and *AABbDD*, and genotypes *AAbbDD* and *AABBDD* are compared using the Student’s *t*-test. The sign * stands for the significant difference at *P* < 0.05.

**Supplementary Figure 3. Phenotypic effects of mutations induced by CRISPR-Cas9 in the *TaGW7* gene in the T3 generation plants derived from YLD2-5-1-52.** **A)** Box and whisker plots show the distribution of grain width, grain length, grain area, and TGW for wild-type and mutant wheat lines. The mean value for each genotype is shown as a red circle. The genotypes of the *TaGW7* homoeologs are shown with lower and uppercase letters corresponding to the mutant and wild-type alleles, respectively, for the A, B, and D genome homoeologs. **B)** The TGW and grain morphometric traits in the T3 generation plants derived from YLD2-5-1-52.

**Supplementary Figure 4. Phenotypic effects of mutations induced by CRISPR-Cas9 in the *TaGW7* gene in the T3 generation plants derived from YLD2-5-1-41.** Box and whisker plots show the distribution of grain width, grain length, grain area, and TGW for wild-type and mutant wheat lines. The mean value for each genotype is shown as a red circle. The genotypes of the *TaGW7* homoeologs are shown with lower and uppercase letters corresponding to the mutant and wild-type alleles, respectively, for the A, B, and D genome homoeologs.

**Supplementary Figure 5. Validation of the *TaGW7* gene homoeolog-specific primers for RT-PCR.** PCR amplification of cv. Chinese Spring (CS) and nullisomic-tetrasomic lines using the genome-specific primer sets: **A)** GW7A_RT_F and GW7A_RT_R2, **B)** GW7B_RT_F and GW7B_RT_R, **C)** GW7D_RT_F and GW7D_RT_R. PCR was performed using DNA isolated from six nullisomic-tetrasomic lines (N2A-T2B, N2A-T2D, N2B-T2A, N2B-T2D, N2DT2A, and N2DT2B), cv. Chinese Spring (CS), and cv. Bobwhite (BW).

**Supplementary Figure 6. The expression levels of the *TaGW7* homoeologs in cv. Azhurnaya and cv. Chinese Spring. A, B)** The expression of the *TaGW7* homoeologs in cv. Azhurnaya was downloaded from wheat eFP Browser. The three homoeologs are represent by three genes TraesCS2A02G176000, TraesCS2B02G202300, and TraesCS2D02G183400 in the wheat eFP Browser. TPM, transcript per million. **C)** The expression data for the *TaGW7* homoeologs in cv. Chinese Spring was downloaded from WheatExp (https://wheat.pw.usda.gov/WheatExp/). The three homoeologs are represented by three transcripts Traes_2AS_2C93BAE62.1, Traes_2BS_79A4D889F.2, and Traes_2DS_1E3DBE64C.1 in the WheatExp website. FPKM, Fragments Per Kilobase Million.

**Supplementary Figure 7. The sequences of pA9ReYFP constructs.**

The construct backbones are pUC19. The maize *ubiquitin* promoter is shown with the lower case letters, the eYFP sequence is highlighted in yellow color, the NOS terminator (termination sequence of the nopaline synthase gene) is underlined and shown in black color. The BamHI, SpeI, KpnI, and MluI cut sites are underlined and shown in blue, purple, red, and green colors, respectively.

**Supplementary Table 1.** The list of primers and oligonucleotides used in the study.

**Supplementary Table 2.** Assessment of gRNA editing efficiency in the wheat protoplasts.

**Supplementary Table 3.** List of genes co-expressed with the *TaGW7* gene homoeologs.

**Supplementary Table 4.** GO terms significantly enriched in the *TaGW7*-associated gene networks.

**Supplementary Table 5.** Wheat genes orthologous to genes in rice that encode proteins interacting with rice GW7 in the yeast two-hybrid (Y2H) assay.

**Supplementary Table 6.** Haplotypes of the *TaGW7* genes identified in hexaploid and tetraploid wheat.
